# Kinetic Characterisation of a Single Chain Antibody against the Hormone Abscisic Acid: Comparison with Its Parental Monoclonal

**DOI:** 10.1371/journal.pone.0152148

**Published:** 2016-03-29

**Authors:** George O. Badescu, Andrew Marsh, Timothy R. Smith, Andrew J. Thompson, Richard M. Napier

**Affiliations:** 1 School of Life Sciences, University of Warwick, Warwick, CV4 7AL, United Kingdom; 2 Department of Chemistry, University of Warwick, Warwick, CV4 7AL, United Kingdom; Naval Research Laboratory, UNITED STATES

## Abstract

A single-chain Fv fragment antibody (scFv) specific for the plant hormone abscisic acid (ABA) has been expressed in the bacterium *Escherichia coli* as a fusion protein. The kinetics of ABA binding have been measured using surface plasmon resonance spectrometry (BIAcore 2000) using surface and solution assays. Care was taken to calculate the concentration of active protein in each sample using initial rate measurements under conditions of partial mass transport limitation. The fusion product, parental monoclonal antibody and the free scFv all have low nanomolar affinity constants, but there is a lower dissociation rate constant for the parental monoclonal resulting in a three-fold greater affinity. Analogue specificity was tested and structure-activity binding preferences measured. The biologically-active (+)-ABA enantiomer is recognised with an affinity three orders of magnitude higher than the inactive (-)-ABA. Metabolites of ABA including phaseic acid, dihydrophaseic acid and deoxy-ABA have affinities over 100-fold lower than that for (+)-ABA. These properties of the scFv make it suitable as a sensor domain in bioreporters specific for the naturally occurring form of ABA.

## Introduction

Antibodies can be harnessed into novel biosensors in order to study the dynamics of their antigens in greater temporal and quantitative detail than has been previously possible. Many biosensors have used enzymes as sensing domains because the enzyme usually has a high specificity for substrate and the progress of the reaction can be quantified relatively easily [1]. For analytes with no accessible enzyme as bioselector, antibodies have offered an alternative sensing domain. The versatility of the immune system to generate antibodies with high affinity and specificity for most analytes, large and small, makes antibodies the bioselector of choice for many applications. The monoclonal antibody hybridoma lines are immortalised and will continue to produce valuable antibodies, but monoclonals are expensive and, for some applications, smaller units are preferred.

Recombinant antibodies such as single chain antibodies generated from fused variable domains (scFv) [2] are versatile tools and may be expressed in some *E*. *coli* lines in good yield. Not all scFvs retain the activity or specificity of the parental monoclonal antibody and so, if they are to be useful in biosensors, it is essential to test the selectivity of the scFv against the parent immunoglobulin and to understand the kinetics of binding.

A number of monoclonal antibodies have been produced against the plant hormone abscisic acid (ABA) including MAC 62 and its subclone MAC 252 [3–5]. These and polyclonals are used widely to measure ABA content in plant material by radiolabelled immunoassays (RIA) or ELISA [4, 6–8] and, increasingly, similar antibody reagents are being used as bioselectors for biosensors [9–11]. Only one of the monoclonals has been sequenced, 15-I-C5 (GenBank CAA82617.1) and a functional, ABA-binding scFv was previously constructed from the variable domains of this monoclonal [12, 13]. When expressed *in planta* it is active and shown to immunomodulate ABA activity in various plant tissues [14, 15], making it ideal for further exploitation.

Plant hormones control growth, development and responses to changing environmental conditions. Biosensors can report such changes *in vivo* and in real time, helping us to improve our understanding of plant biology. Two biosensors for ABA have been reported recently, both based on fusions of the plant ABA receptor protein, its interaction partner and a pair of fluorescent proteins to give a Förster resonance energy transfer cassette. These have affinities for ABA ranging from around 0.1 μM [16] to 80 μM [17] and they function well *in planta* to report changes in ABA concentration. However, antibodies can offer greater sensitivity (higher affinity), might give options for sensor modules which do not conflict with normal receptor interactions, and can be adapted for e.g. high-throughput *ex vivo* measurements. Consequently, we have examined the suitability of an anti–ABA scFv which can be readily prepared from bacterial expression cultures.

Immunogloblin structure and activity depends greatly on structure-stabilising disulphide bonds between conserved cysteine residues. The formation of these disulfide bonds usually occurs co-translationally in the oxidising environment of the mammalian endoplasmic reticulum, although they may also form in the periplasm of Gram-negative bacteria [18]. However, protein yields from periplasmic expression are small compared to cytoplasmic expression. Recombinant scFv expressed in the cytoplasm may not form disulfide bonds [19, 20] and tend to aggregate as insoluble inclusion bodies of unfolded protein, but active scFv can be recovered from cell lines carrying mutations in thioredoxin pathway genes (*trxB* and *gor*) [20]. Solubility can also be improved by creating fusions to highly soluble partners and maltose-binding protein (MBP) in particular has been reported to act as a good folding scaffold for active scFvs in *E*. *coli* [21–23]. The MBP fusion also facilitates purification under mild conditions. In this paper we describe the activity and kinetics of 15-I-C5 and its derivative, MBP-antiABA-scFv, using both heterogeneous phase (solution–solid surface) assays with a surface plasmon resonance biosensor (steady-state SPR) and a homogeneous (solution) phase SPR technique using competitive titration.

## Materials and Methods

### Synthetic Gene for Recombinant Antibody

The polypeptide sequence of the mouse anti-ABA scFV (GenBank CAA82617.1) was input to DNAWorks [24] to design 40 oligonucleotides which assemble into a DNA sequence with codon optimisation for *E*. *coli* expression (based on *E*. *coli* class II genes). A HindIII cloning site was added to the DNA sequence at the amino terminus, and a NotI site at the carboxy terminus, of the encoded protein. The oligonucleotides were assembled by PCR [24] using high-fidelity DNA polymerase (UltraPfu, Stratagene), and the PCR product was cloned into the HindIII and NotI sites of pBluescriptII (Stratagene). A clone with the correct DNA sequence was confirmed by dideoxy sequencing (GenBank accession KU170610); the encoded amino acid sequence remains identical to CAA82617.1. Additional methods are described in S1 Additional Methods).

Using a directional cloning strategy the DNA was cloned into the pMal system (New England Biolabs, UK) resulting in the expression of an N-terminal MBP fusion protein (MBP-antiABA-scFv). The synthetic gene was amplified using PCR primers S1 Additional Methods and cloned into the multiple cloning polylinker of the pMal vector. The recombinant plasmid was transformed into DH5ά competent cells and resulting colonies were screened using blue/white selection. Positive colonies were analysed by PCR and restriction digest for presence of the insert. Plasmids containing an insert of the predicted size were sequenced (insert and flanking vector). The recombinant vector carrying the correct sequence for antiABA scFv in-frame with malE was transformed into Origami B, Rossetta-gami B and BL21 competent cells. The pMal vectors use the strong Ptac promoter and the *malE* translation initiation signal to give high-levels of expression and the Lac repressor gene *laclq*, which keeps expression low in the absence of IPTG. The p2x version contains the intact signal sequence of the *malE* gene, allowing the fusion proteins to be exported to the periplasmic space (S1 Fig). The c2x version has a deletion of the signal sequence of *malE*, leading to cytoplasmic expression of the fusion protein. Plasmids from selected recombinants containing an insert of the predicted size were sequenced (insert and flanking vector) and correct, in-frame vector was transformed into Origami B, Rossetta-gami B and BL21 cell lines.

### Protein Production

Cells were cultured on Rich-Broth medium with ampicillin selection (recommended in the pMal manual) or Lysogeny broth (LB) with ampicillin + 0.4 M sucrose [25]. When cells were cultured on Rich-Broth there was copious expression of free MBP even without induction. When cells were cultured on LB + ampicillin + 100 mM glucose, no expression of the free MBP could be detected in uninduced cells, indicating that 100 mM glucose was enough to suppress expression from the tac promoter. Isopropyl β-D-1-thiogalactopyranoside (IPTG) induction (0.05–2 mM at 16–37°C for 2–24 hours) of cells cultured on LB + ampicillin + 0.4 M sucrose resulted in a better yield of the fusion protein. Expressed soluble proteins were harvested from the periplasm using cold osmotic shock, cytoplasmic proteins by lysis using sonication. In each case the scFv was purified by affinity chromatography using amylose-linked Sepharose (New England Biolabs, UK) and elution with 10 mM maltose. A table of yields is given in S1 Table.

### Release of antiABA-scFv from the Fusion Protein

The pMal-p2x vector includes a Factor Xa protease sequence allowing the scFv to be cleaved from MBP after purification. The fusion protein was incubated at 20°C for 16 hours with Factor Xa (New England Biolabs, UK) and the products separated by HiTrapQ (GE Healthcare, UK) anion exchange chromatography eluted with a gradient of NaCl (0 to 0.5 M). A linear gradient resulted in poor separation (the peaks were overlapping) and so, as each protein peak started to elute, the NaCl concentration was held until the signal returned to baseline. This method allowed complete separation of the free scFv from both MBP and the uncleaved fusion protein.

### ABA Binding Activity

Antibody in buffer (20 mM sodium citrate, 5 mM MgSO_4_, pH 6.0) was mixed with 150 Bq DL-*cis*,*trans*-[H]-abscisic acid ([3H]-(+/-)-ABA; Amersham, GE Healthcare) and 125 μg bovine γ-globulin, +/- saturating (10^−5^ M) cold (+)-ABA, all in 100 μL. All assays were in triplicate. After incubation for one hour at 4°C in darkness, three volumes of saturated ammonium sulphate was added followed by a further 15 min incubation on ice. Protein was pelleted by centrifugation, pellets rinsed carefully with saturated ammonium sulphate before solubilisation in EcoScint scintillation fluid (National Diagnostics) and radioactivity measured by scintillation counting (Beckman-LS6000TA).

### Surface Plasmon Resonance

This study used a BIAcore 2000 (Biacore, GE Healthcare). Biacore streptavidin (SA) and carboxy-activated (CM5) chips, buffers and reagents were purchased from GE Healthcare, UK. If buffers were prepared locally, they were filtered (0.2 micron) and degassed before use.

### Synthesis of Biotinylated ABA

*S*-(+)-abscisic acid 4’-(*N*-succinamidyl acyl hydrazide-*N*’-biotinyl-3,6-dioxaoctane-1,8 diamine) (S2 Fig) and 2-*cis*-(*R*)-(+)-Abscisic acid: (2-*cis*-(*R*)-(+)-ABA) were prepared as previously described [26].

### Commercial anti-ABA Monoclonal Antibodies

Monoclonal anti-ABA MAC 252 52 [3] was purchased from Abcam (product no ab50594). Monoclonal anti-ABA 15-I-C5 [4] was purchased from Biofords Consultants SARL (Agdia, Evry Genopole, France) as Phytodetek ABA-15-I-C5 Monoclonal Antibody.

## Results and Discussion

### Activity of MBP-antiABA-scFv after Expression in *E*. *coli*

Immunological reagents are widely used analytical tools. For the phytohormone ABA both monoclonal and polyclonal reagents are available, but scFvs offer the advantages of e.g. cost-effective, reproducible production using bacterial expression. Therefore, we developed a scFv from the sequence of one of the widely used anti-ABA monoclonals. Some antibodies and scFvs are compromised by expression in *E*. *coli* (e.g. [27]) and so the characteristics of the scFv have been determined.

Expression was found to be optimal after growing cells at 37°C in the presence of 100 mM glucose to an A_600_ of approximately 0.5, followed by mild and slow induction with 20 μM IPTG in the presence of 0.4 M sucrose at 18°C for 16 hours. However, the yield of fusion protein recovered from the periplasm was low (about 0.5 mg/L; S1 Table). Cytoplasmic expression using the pMal system usually yields considerably more protein than when the same protein is exported into the periplasmic space, but there can be problems with folding. Nevertheless, successful cytoplasmic expression of soluble scFvs has been reported in *E*. *coli* cells carrying mutations in thioredoxin pathway genes (such as Rosetta-gami and Origami cells; 27). Recovering both the periplasmic and cytoplasmic proteins by sonicating Rosetta-gami B cells carrying the pAB1 plasmid yielded in excess of 20 mg/L of amylose-binding soluble fusion protein (Origami cells 21.6 mg/L) representing about 10% of the total soluble protein.

The activity of the purified MBP-antiABA-scFv product (for brevity we will refer to this as scFv hereafter) was assessed in a radiolabel binding assay using [3H]-(+/-)-ABA (S3 Fig). The data showed that the scFv was active, and displayed a nanomolar affinity for free ABA at physiological pH. Binding was found to be pH-independent between pH 5.5 and pH 8.5, but fell off sharply below pH 5.5 as the ligand approached its pKa (pKa ABA = 4.75). For more detailed, kinetic characterisation of the scFv, surface plasmon resonance (SPR) was used.

### BIAcore Parameterisation

Instruments based on SPR such as BIAcore offer some of the most direct and reliable methods for determining both association and dissociation kinetic rate constants [28, 29]. Ideally, in order to minimize mass transport limitation (MTL), a low level of ligand immobilisation is required, such as to give maximal analyte binding (R_max_) of about 100 resonance units (RU) [30, 31]. This required loading 1 RU of the biotinylated ligand b-PEG-ABA onto an SA chip, which proved technically challenging. As an alternative, three parallel channels of a CM5 chip were coated with different streptavidin levels (approximately 90 RU, 550 RU and 3350 RU). Biocytin was bound to flow cell one for use as the in-line reference, channels 2, 3 and 4 were saturated with b-PEG-ABA. The homogeneity of the prepared surfaces was verified by monitoring the output from the θ_SPR_ photo-detector array.

When scFv was injected over the chip surface specific binding was observed. This binding competed away by mixing scFv in solution with excess free ligand, 1 μM (+)-ABA. Regeneration of the chip surfaces was achieved with a 1 min pulse of 50 mM NaOH. The performance of the sensor chip surface with bound b-PEG-ABA was assessed by monitoring repeated binding and regeneration cycles. No significant build-up of protein on the chip surface or loss of binding capacity was observed after 36 regeneration cycles.

### Quantitation of the Analyte

**Fig 1:**
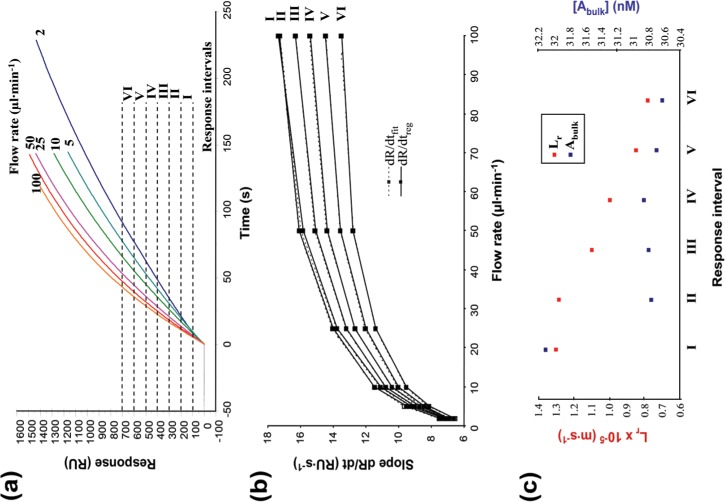
Calculating the concentration of active scFv. The scFv (142 nM, as determined by measuring the optical density at 280nm) was injected over a chip surface with high ligand immobilisation at several flow rates. (a) The association phase of the sensorgrams was divided into six response intervals (I-VI). (b) Comparison of the experimental (dR/dtreg) and calculated (dR/dtfit) binding rates for each response interval. (c) Lr (red) and [Abulk] (blue) were calculated by global fitting of the data at each flow rate using Eq 2, using dR/dtreg as slopes for each response interval. Lr values decrease with the increase of the response interval, while the calculated [Abulk] values are contained in a small interval (30.622–32.098 nM). The calculated concentration of active scFv is the average [Abulk] from each response interval: 30.97 ± 0.559 nM (SD = 1.8%).

Determination of affinity and association rate constants needs the concentration of active analyte to be known with great precision. Bacterial expression can yield both active and inactive (unfolded etc.) scFv, and so the method developed by Christensen [32] was used to determine the concentration of active analyte. Purified scFv (nominal protein concentration 142 nM/10.57 μg/ml, determined by A_280_) was injected over channel 4 with high ligand density (R_max_ ≈ 3100 RU), at several flow rates (2, 5, 10, 25, 50 and 100 μl/min). The association phase of each sensorgram was divided into six response intervals (Fig 1). The slopes of the curves (dR/dt_reg_) were determined for each response interval by linear regression at each flow rate giving low χ^2^ values (χ^2^_reg_) ranging between 0 and 0.21 (S2 Table). The mass transport coefficient (L_m_ in m∙s^-1^) was calculated according to Eq 1.

$$L_{m}=C_{Lm}\sqrt[{3}]{\frac{D^{2}\cdot F}{h^{2}\cdot b\cdot l}}$$ Equation 1

where *C*_*Lm*_ has a value of 1.21 for a BIAcore 2000 instrument with detection between 0.4 and 2.0 mm, l = 1.6 mm, D is the analyte diffusion coefficient D estimated using Stoke’s law and the Einstein-Sutherland equation, F is the flow rate, h is the height of the flow cell, b the width and l the length.

The concentration of active scFv (A_bulk_) and the coefficient of reaction flux (L_r_) were determined by globally fitting the data to Eq 2 at each flow rate, using dR/dt_reg_ as slopes for each response interval.

$$\frac{dR}{dt}=\frac{L_{m}\cdot M_{r}\cdot G\cdot L_{r}[A_{bulk}]}{L_{m}+L_{r}}$$ Equation 2

where R is the response level (RU) and G is a parameter that corresponds to the signal obtained when 1 ng of protein is captured over a 1mm^2^ surface of the dextran matrix (1000 RU mm^2^∙ng^-1^). Very good fits were obtained (χ^2^_fit_ = 0.01–0.018). Introducing the calculated L_m_, L_r_ and A_bulk_ concentrations into Eq 2 gave simulations of ideal slopes (dR/dt_fit_), and these fitted closely to the experimental slopes (dR/dt_reg_) (Fig 1B). As the binding proceeds during sample injection, an increasing number of binding sites on the chip surface become occupied, reducing the ligand density, which in turn reduces the flux of the binding reaction. This is indicated by the observed decrease of the L_r_ value with the later and increasing response intervals (Fig 1C).

A low variation (SD = 1.8%) of the A_bulk_ concentrations determined in each response interval was observed (Fig 1C) indicating that the measurements are accurate and reliable. The concentration of active scFv was calculated to be 31.0 ± 0.56 nM, the mean of the A_bulk_ values determined in each response interval, indicating that only 21.8% of amylose-purified MBP-antiABA-scFv was active. This suggests that a large proportion of the protein is misfolded or damaged, but by knowing the concentration of active scFv it was possible to progress to kinetic assays without the need for further purification. The same method was used to determine the concentrations of active antibody for subsequent experiments with cleaved scFv (MBP removed) and the parental monoclonal 15-I-C5 (S3 Table). By way of verification, the active concentrations of selected samples were also estimated by the method of Kazemeir [33] which gave very similar answers (S4 Fig).

### Kinetic and Affinity Constants

**Fig 2:**
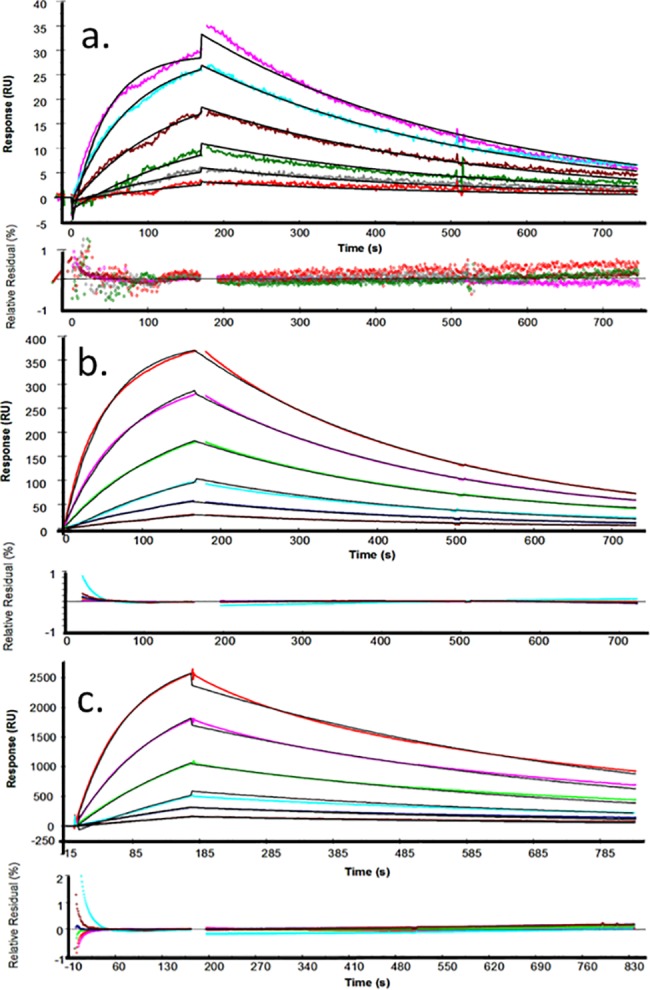
Kinetic measurements on surfaces with different ligand densities. Six concentrations of scFv (2-fold dilutions from 24 to 0.75 nM) were injected at 25°C. The responses from the in-line reference flow cell and blank injections were subtracted from all curves. The curves were globally fitted to a 1:1 Langmuir binding model. For each surface density, the experimental sensorgrams (coloured lines) were overlain with the theoretical fitted curves (black lines). In each case, the relative residual plots show the difference between the experimental and the theoretical curves expressed as a percentage of the observed response for each analyte concentration. The spikes observed at the beginning and the end of analyte injections are the result of in-line reference subtraction with the slight sensorgran misalignment introduced with sequential analyte flow in the multichannel mode. Nevertheless, residuals are mostly within 1% of R_obs_. The surface density, expressed as the maximum analyte binding capacity (Rmax) was determined experimentally: Rmax ≈ 49 RU (a); 465 RU (b); 3100 RU (c).

In order to obtain reliable kinetic data we optimised the system and tested for MTL [31]. All injections were carried out in the multichannel mode at 25°C at high flow rate (50 μl/min) and at scFv concentrations ranging from 24 to 0.75 nM. The response from the in-line reference (channel one), as well as the response from blank injections of buffer, were subtracted from all sensorgrams (double referencing). The sensorgrams were globally fitted (BIAevaluation 4.1) using a simultaneous *k*_*a*_*/k*_*d*_ 1:1 Langmuir binding model (Fig 2). The residual plots show no significant systematic deviations for the low density surface. Small deviations were observed on medium and high ligand densities, indicating that binding was mass transport limited on these surfaces. As a further check, the data were fitted to a 1:1 Langmuir binding model that included a mass transport parameter (S5 Fig). Similar rate constants were obtained from fitting the data from low density surface to both binding models (1:1 and 1:1 with MTL), suggesting that the binding to this surface is not mass transport limited. Fitting the sensorgrams obtained on the medium density surface to the 1:1 binding model with MTL gave a small underestimation, but the model failed to compensate for the much higher levels of MTL observed on the high density surface. All the data are summarised in S4 Table.

The measured *K*_*D*_ for this recombinant antibody purified from the periplasm is (3.6 ± 0.27) x 10^−9^ M. Values calculated for *K*_*a*_ [(8.7 ± 0.29) x 10^5^ M^-1^∙s^-1^] and *K*_*d*_ [(3.1 ± 0.06) x 10^−3^ s^-1^] vary little between methods and values shown are from use of the Langmuir 1:1 model. Using the same model, values for the scFv purified from the cytoplasm are similar (*K*_*D*_ = 2.8 x 10^−9^ M, χ^2^ = 0.621; *K*_a =_ 9.3 ± 0.38 x 10^5^ M^-1^s^-1^ and *K*_*d*_ = 2.6 ± 0.07 x 10^−3^ s^-1^; S4A Table). Consequently, we no longer treat scFv purified from periplasm and cytoplasm separately.

All heterogeneous phase (surface) techniques can be subject to artefacts introduced by ligand immobilisation or e.g. biotinylation of the free ligand. Therefore, affinity in solution was determined by mixing scFv (25 nM) with different concentrations of (+)-ABA (0.5, 1, 5, 10, 50, 100, 200 and 500 nM) before injection. The equilibrium mixtures (1 hour at 25°C) were injected over an SA chip surface saturated with b-PEG-ABA at high ligand density, and the initial binding rate of free scFv was measured. The concentration of free scFv from each equilibrium mixture was determined from a calibration curve (S6A Fig) and plotted against the concentration of free (+)-ABA. Data points were fitted to the solution affinity model in BIAevaluation (S5B Fig). A good fit (χ^2^ = 1.01 x 10^−19^) with low residuals (within ± 0.2%) gave a solution affinity constant (K_D_ = 3.4 x 10^−9^ M) in good agreement with the value determined by surface techniques (Table 1). Thus, the kinetics of the fusion protein have been described with confidence. Next these were compared with the characteristics of both cleaved, free scFv and with the parental monoclonal immunoglobulin.

**Table 1:** Comparing values of affinity constant determined by surface (kinetic and equilibrium) and solution (equilibrium) assays for the scFv.

	Method		
**Parameter**	Kinetic analysis	Steady-state binding (reverse orientation)	Affinity in solution
**Ligand**	Immobilised b-PEG-ABA	Immobilised MBP-aABA-scFv	Free (+)-ABA
**Analyte**	Free MBP-aABA-scFv	Free b-PEG-ABA	Free MBP-aABA-scFv
**K**_**D**_ **(nM)**	3.55±0.27	3.70	3.37
**χ**^**2**^	0.19	0.065	1.01E-19

### Cleaved (Free) antiABA-scFv

AntiABA-scFv was successfully cleaved from the MBP fusion protein by digestion with Factor Xa and purified by ion exchange chromatography. The active concentration of free scFv was determined by SPR, as above, and serial dilutions (40–1.25 nM) were injected over low density b-PEG-ABA and control channels. The sensorgrams were globally fitted to a 1:1 Langmuir binding model (*χ*^*2*^ = 0.141). The kinetic constants and affinity (*K*_*D*_ = 3.2 x 10^−9^ M) were in good agreement to values calculated for the fusion MBP-antiABA-scFv (S7 Fig, S5B Table). The presence of the C-terminal MBP fusion protein had no measurable effect on the activity of the scFv.

### 15-I-C5, the Parental Monoclonal Antibody

The binding properties of the parental monoclonal antibody (15-I-C5) were also evaluated. Sequential injections of 2 nM 15-I-C5 were made over low density b-PEG-ABA and control sensor chip surfaces (S7 Fig). The apparent association rate constant of 15-I-C5 was faster and the apparent dissociation rate constant considerably slower than for the scFv. However, immunogobulins are bivalent and when sensorgrams were fitted to a bivalent binding model the calculated association immobilised b-PEG-ABA was very close to the values measured for MBP-antiABA-rate constant for the modelled monovalent interaction between 15-I-C5 and scFv and the free scFv (Table 2). The dissociation rate constant was about 2.5 fold slower, resulting in a higher affinity (lower *K*_*D*_) for the parental immunoglobulin than the scFv.

**Table 2:** Kinetic and affinity constants for 15-I-C5.

Parameter	Kinetic analysis		Affinity in solution
	1:1 Langmuir	Bivalent analyte	
*k*_*a1*_ (M^-1^∙s^-1^)	3.80E+06	9.55E+05	-
SE (*k*_*a1*_)	2.37E+04	2.01E+05	-
*k*_*d1*_ (s^-1^)	5.59E-06	1.18E-03	-
SE (*k*_*d1*_)	4.87E-06	2.95E-03	-
*k*_*a2*_ (s^-1^)	-	0.0245	-
SE (*k*_*a2*_)	-	0.0474	-
*k*_*d2*_ (s^-1^)	-	6.61E-04	-
SE (*k*_*d2*_)	-	1.41E-03	-
*K*_*D*_ (M)	1.47E-12	1.23E-09*	1.13E-09
*χ*^*2*^	0.152	0.152	-

*The affinity of the modelled monovalent component of the interaction was calculated from *K*_*D*_ = *k*_*d1*_*/k*_*a1*_. SE, standard error based on each global fit in BIAevaluation. The sensorgrams are shown in S8 Fig.

**Fig 3:**
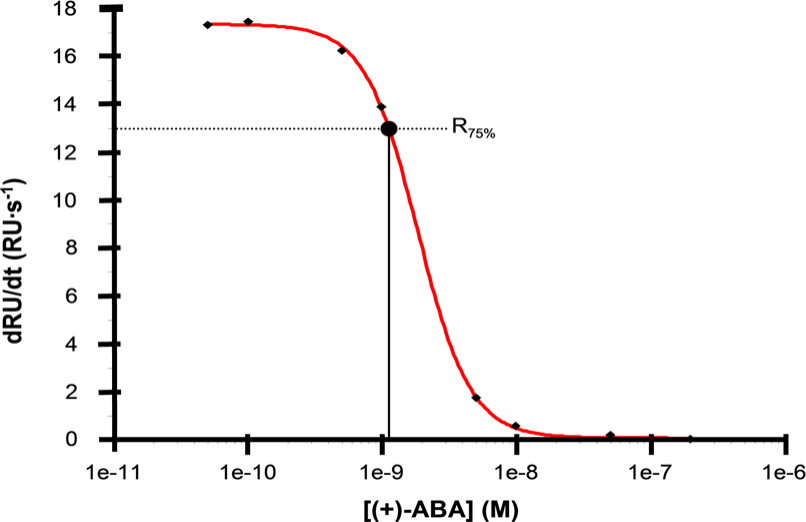
Affinity in solution 15-I-C5. Monoclonal 15-I-C5 (5 nM) was mixed with different concentrations of (+)-ABA (0.5–500 nM) for 1 hour at 25°C. The equilibrium mixtures were injected over a sensor chip surface with high b-PEG-ABA density and the initial binding slopes were measured for each sample. The values were plotted against the concentration of free ABA and the data points were fitted to a 4-parameter equation. KD was estimated from the corrected inflection midpoint of the fitted curve (Stöcklein et al., 1998). The affinity in solution of 15-I-C5 for (+)-ABA was estimated to 1.13 nM.

In contrast to the direct, surface binding experiments, avidity should not affect the binding of a small antigen in solution. The modelled kinetics were validated by testing in solution (Fig 3), as above. The affinity in solution (*K*_*D*_) was estimated [34] for (+)-ABA to be 1.13 nM, in agreement with the value determined by surface binding kinetics (Table 2). The high affinities illustrate one reason why this monoclonal became widely adopted for ABA assays from plant tissue extracts.

### Selectivity of MBP-antiABA-scFv

**Fig 4:**
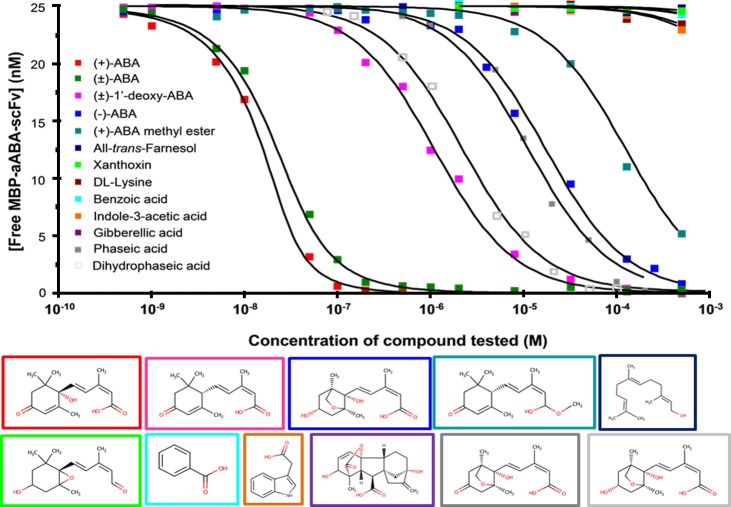
Cross-reactivity of the anti-ABA-scFv. MBP-antiABA-scFv (25 nM) was mixed with different concentrations of each test compound for 1 hour at 25°C. The concentration of free scFv in the equilibrium mixtures was plotted against the concentration of test compound. The structures of each competitor are shown framed in the same colours as the data in the dose graph. There is no box for the racemic mix. The colour code for each compound is shown on the left of the plot.

The selectivity of the scFv for (+)-ABA was tested by determining the affinity in solution for structurally or physiologically related compounds. The scFv (25 nM) was mixed with different concentrations of test compound and incubated for 1 hour at 25°C. The concentration of free scFv in the equilibrium mixtures was determined and plotted against concentration of competitor. Affinity was determined by fitting the data points to a solution affinity model (Fig 4). The *K*_*A*_ value was determined for each compound, and then percentage cross-reactivity was calculated by expressing each *K*_*A*_ value relative to the *K*_*A*_ for (+)-ABA. Values were compared to literature data for the parental monoclonal antibody (Table 3).

**Table 3:** Selectivity of MBP-aABA-scFv.

Compound	Affinity in solution, MBP-aABA-scFv			Cross-reactivity MBP-aABA-scFv (%) ^a^	Cross-reactivity mAb 15-I-C5 (%) ^b^
	K_D_ (M)	SE (K_D_)	K_A_ (M^-1^)		
2-*cis*-(S)-(+)-ABA	3.37E-09	7.43E-11	2.96E+08	100	100
2-*cis*-(R,S)-(±)-ABA	7.61E-09	1.08E-10	1.31E+08	44.25	49.4 ^d^
2-*cis*-(R)-(-)-ABA	1.73E-05	1.36E-07	5.71E+04	0.019	0 ^c^
2-*cis*-(R,S)-(±)-1’-deoxy-ABA	1.09E-06	4.29E-08	9.17E+05	0.309	n.r.
2-*cis*-(S)-(+)-ABA methyl ester	1.21E-05	6.04E-07	8.26E+04	0.028	<0.1 ^d^
2-*trans*-(S)-(+)-ABA	n.t.	-	-	-	0 ^c^, 0.98 ^d^
Phaseic acid	1.13E-05	1.16E-06	8.82E+04	0.029	<0.1 ^c^
Dihydrophaseic acid	2.17E-06	5.18E-07	4.62E+05	0.156	<0.1 ^c^
All-*trans*-Farnesol	1.71E-02	5.06E-03	5.84E+01	0.00002	0 ^c^
Xanthoxin	1.62E-03	1.09E-04	6.17E+02	0.0002	0 ^c^
*D*,*L*-Lysine	6.12E-03	1.78E-03	1.63E+02	0.00006	n.r.
Indole-3-acetic acid	7.23E-03	9.07E-04	1.38E+02	0.00005	n.r.
Gibberellic acid	3.28E-02	1.50E-03	3.04E+01	0.00001	n.r.
Benzoic acid	1.09E-02	1.33E-03	9.17E+01	0.00003	n.r.

^**a**^ Cross-reactivity was calculated using the *K*_*A*_ value determined for each compound and assigning (+)-ABA the relative value of 100%.

^**b**^ Determined by other investigators from radiolabel displacement curves using 50% displacement.

^**c**^ Reported by manufacturers (Phytodetek manual m100.2, Agdia Inc., Indiana, USA).

^**d**^ Walker-Simmons *et al*., 1991. **n.t.—**not tested. **n.r.**–not reported.

The scFv exhibited a high degree of selectivity for the physiologically active (+)-ABA. A 5000-fold lower cross-reactivity was observed with the optical isomer (-)-ABA, showing that the scFv was able to distinguish sharply between the natural and unnatural (synthetic) ABA enantiomers. Hence, it retained the strict configuration requirements at the chiral centre, position C-1ʹ for (+)-ABA [8]. Compared to pure (+)-ABA, the cross-reactivity with racemic (±)-ABA was a little lower (44.3%) than the anticipated 50%, most likely because the proportion of the two enantiomers in the commercial mixture is not equal. Very low cross-reactivities were observed with ABA-methyl ester (0.028%) and 1ʹ-deoxy-ABA (0.3%), suggesting that the scFv also retained the strict structural requirements of the parental immunoglobulin for the ABA side chain, from the C-1 to C-1ʹ and the free carboxyl group. Very low cross-reactivity was displayed against the early catabolic product of ABA, phaseic acid (0.03%), and for the subsequent catabolite dihydrophaseic acid (0.16%). Almost no cross-reactivity (lower than 10^−4^%) was observed with the ABA precursor xanthoxin, benzoic acid (a control weak organic acid), farnesol (a physiologically-related compound), lysine (a control polar amino-acid present in the plant cell in millimolar concentrations), nor other plant hormones (auxin and gibberellic acid).

## Conclusions

Careful determination of the kinetic properties of a bacterially-expressed scFv has shown it to retain the nanomolar K_D_ characteristic of its parental monoclonal IgG. The values compare favourably with a similar scFv based on 15-I-C5 and expressed in plant leaves, for which an ELISA IC50 value of approx. 1.5 nM was estimated [13]. The selectivity of our purified, bacterial scFv has been determined by quantitative assay, showing it to have a high fidelity for the bioactive (+)-ABA enantiomer and very low cross-reactivity with structurally-related metabolites. Collectively these features suggest that this antiABA-scFv is suitable for assaying this essential hormone in plant extracts and will be an effective and reliable bioselector element for deployment in ABA biosensors.

## Supporting Information

S1 Additional Methods Details of primers and PCR conditions. (PDF)

S1 Fig Schematic representation of the pMal vectors. (PDF)

S2 Fig Biotinylated ABA conjugate, b-PEG-ABA. (PDF)

S3 Fig Radiolabel assay of purified scFv. (PDF)

S4 Fig Calibration curve for active protein concentration (Kazemier method). (PDF)

S5 Fig Checking for mass transport limitation; Simulation of the kinetic constants determined on sensor chip surfaces with different ligand densities. (PDF)

S6 Fig Affinity in solution. (PDF)

S7 Fig Kinetic analysis of free scFv according to different interaction models. (PDF)

S8 Fig Kinetic analysis of the parental monoclonal immunogobulin 15-I-C5. (PDF)

S1 Table Yields from expression and purification of anti-ABA scFv in *E*. *coli*. (PDF)

S2 Table Determination of the concentration of active MBP-antiABA-scFv. (PDF)

S3 Table Determination of the concentration of active proteins by the method of Christensen (1997). (PDF)

S4 Table Kinetic and affinity constants of the scFv determined using alternative kinetic models in BIAevaluation. (PDF)

S5 Table Kinetic and equilibrium constants for the scFv expressed and purified from the cytoplasm of E. coli and of free scFv (fusion protein removed). S5a, Kinetic and equilibrium constants calculated of scFv from cytoplasmic expression in E.coli. S5b. Kinetic and affinity constants calculated for free scFv.
(PDF)
