# Supplementary material for: Kinetic Characterisation of a Single Chain Antibody against the Hormone Abscisic Acid: Comparison with Its Parental Monoclonal
Source: PLoS One. 2016 Mar 29;11(3):e0152148. doi: 10.1371/journal.pone.0152148 (PMC4811560; doi:10.1371/journal.pone.0152148)
Supplement: S1 Additional Methods — (PDF) [file pone.0152148.s001.pdf]

Supplementary data and figures

Additional methods: Details of primers and PCR conditions.

Polymerase chain reaction (PCR)

When the PCR products were intended for cloning or sequencing, proof-reading DNA polymerase PfuUltraTM Hotstart High-Fidelity DNA Polymerase (Stratagene) or Pwo DNA polymerase (Roche) was used. For other applications, standard Taq DNA polymerase (Roche). A typical PCR reaction contained 200 µM of each dNTP, 300 nM of each primer, 1 ng template plasmid DNA, 1X PCR buffer with 2 mM $MgSO_4$, 2.5 U of DNA polymerase and sterile double distilled water to 50 µl. The reaction mixtures were incubated for 5 min at 94ºC to denature DNA and then 25 cycles of the following: 94ºC for 1 min, 50-65ºC for 1 min for annealing, and 72ºC for 1 min for extension. Upon completion the reaction was held at 72ºC for 7min. The annealing temperature depended on the melting temperatures of the primers used. PCR products were analysed by agarose gel electrophoresis.

Primer list:

Primers were designed and analysed using PrimerSelect DNAStar (DNAStar Inc, Madison, USA) or Vector NTI 9 (Invitrogen) software. Primers were obtained from MWG Biotech or Invitrogen**.**

Anti ABA-scFv forward	5'-TCAGAATTCCAGGTTCAGCAGCAGTC-3'
Anti ABA-scFv reverse	5'-TGCTAAGCTTACGCCGCACGTTTCAGTT-3'
Sense aABA scFv 6His	5'-CAATAACAACAACCTCGGGATCGAG-3'
Antisense aABA scFv 6His	5'-CTAATGAAGCTTAATGATGATGATGATGAT GCGCCGCACGTTTCAGTTCCA-3'
pMal pIII sec primer	5'-CACTTCACCAACAAGGACCA-3'
Sense BAP EcoRI for pMal c2x	5'-GGAGCAGGCGAATTCGTGAGCAAG GGCGAGGAGCTGTTCACCGGG-3'
Antisense BAP EcoRI for pMal c2x	5'-GGAGCTGCAAGCTTTTACTCGTGCC ACTCGATCTTCTGGGCCTCGAAGATGTCGTT CAGGCCGCCGCCGG-3'

PCR products were purified using QIAquick PCR purification kit (Qiagen) according to the manufacturers' instructions.
