# Supplementary material for: Kinetic Characterisation of a Single Chain Antibody against the Hormone Abscisic Acid: Comparison with Its Parental Monoclonal
Source: PLoS One. 2016 Mar 29;11(3):e0152148. doi: 10.1371/journal.pone.0152148 (PMC4811560; doi:10.1371/journal.pone.0152148)

Figure S1. Schematic representation of the pMal vector. (a) pMal p2x vector with anti-ABA scFv. The pMal c2x version of the vector has a deletion of the signal sequence of malE, leading to cytoplasmic expression of the fusion protein. (b) Schematic representation of the polylinker of pMal-p2x and pMal-c2x. The protease (Factor Xa) cleavage site and unique restriction sites are indicated.

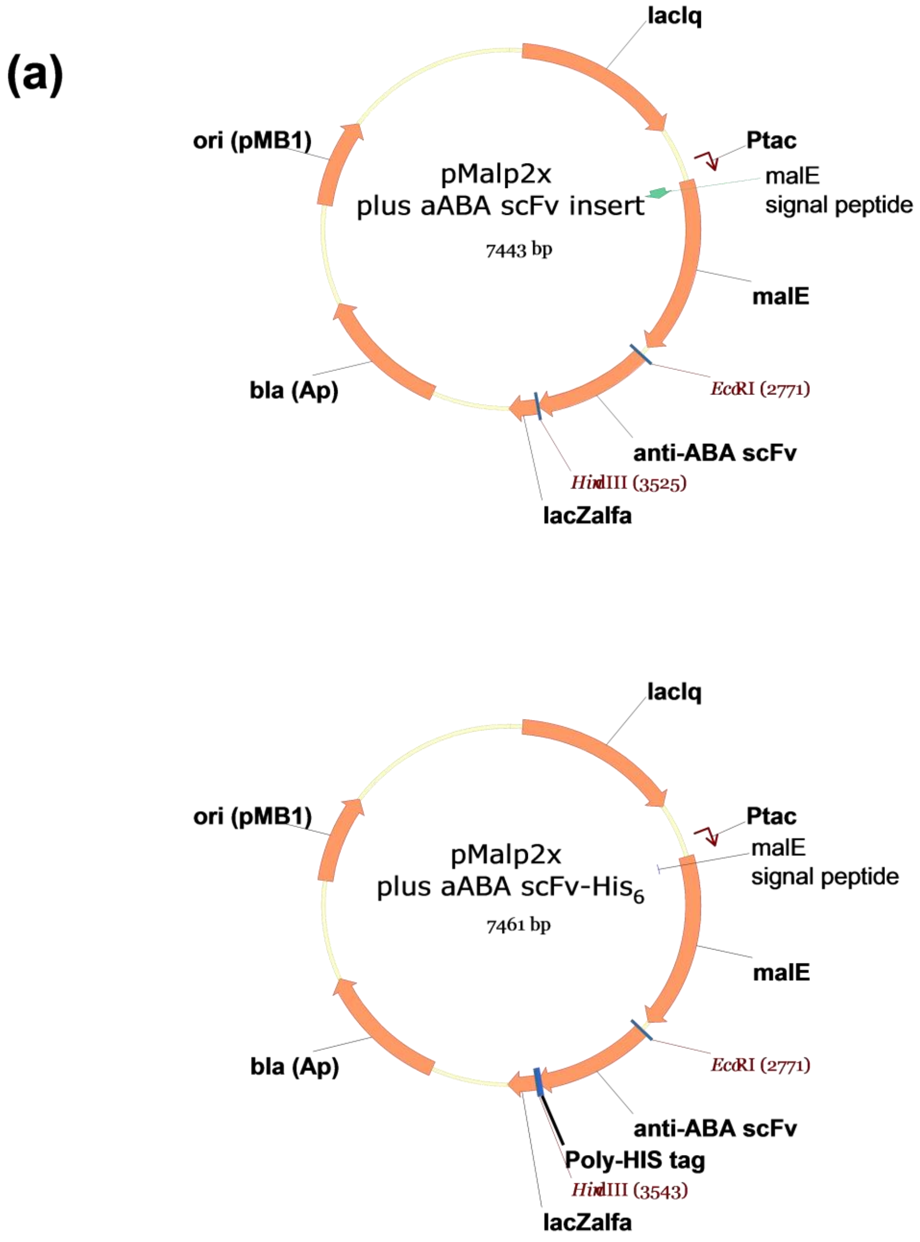

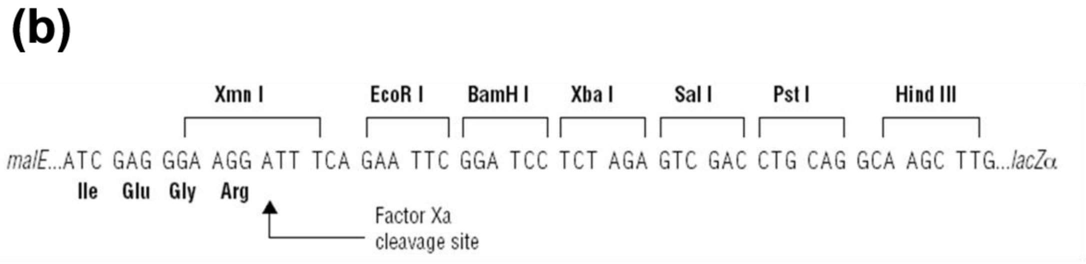

Supplement: S1 Fig — (PDF) [file pone.0152148.s002.pdf]
