# Supplementary figures and images for: Kinetic Characterisation of a Single Chain Antibody against the Hormone Abscisic Acid: Comparison with Its Parental Monoclonal
Source: PLoS One. 2016 Mar 29;11(3):e0152148. doi: 10.1371/journal.pone.0152148 (PMC4811560; doi:10.1371/journal.pone.0152148)

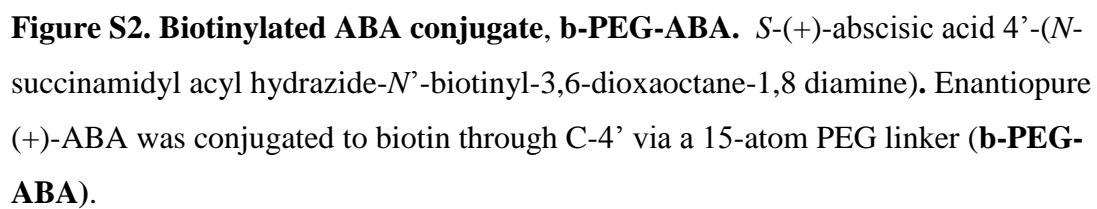

Supplement: S2 Fig — (PDF) [file pone.0152148.s003.pdf]
