# Supplementary material for: Kinetic Characterisation of a Single Chain Antibody against the Hormone Abscisic Acid: Comparison with Its Parental Monoclonal
Source: PLoS One. 2016 Mar 29;11(3):e0152148. doi: 10.1371/journal.pone.0152148 (PMC4811560; doi:10.1371/journal.pone.0152148)
Supplement: S3 Fig — (PDF) [file pone.0152148.s004.pdf]

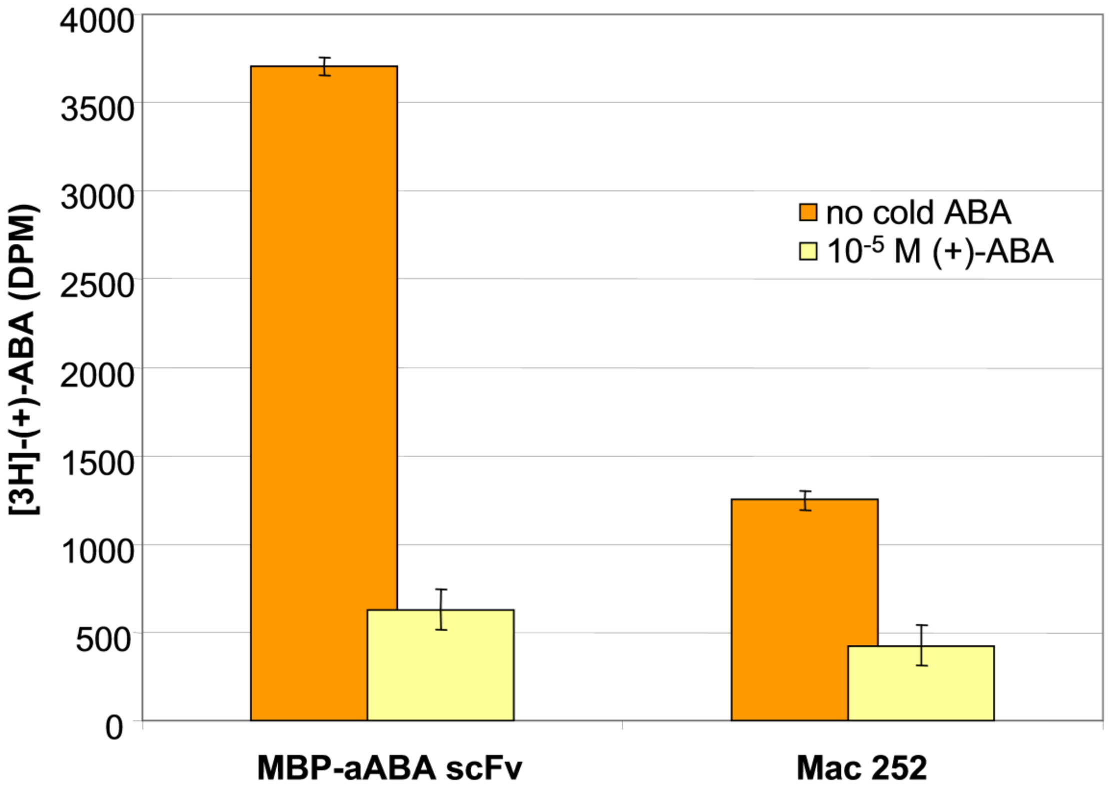

Figure S3: Radiolabel assay of purified scFv. MAC252, a commercially available monoclonal for ABA, was used as a positive control (diluted 1:5,000). The fusion protein was incubated with $[^3H]$-ABA +/- 10^{-5}M (+)-ABA (cold ABA) for 1h at 4ºC. Bound $[^3H]$ABA was precipitated with protein using 50% saturated ammonium sulphate. Saturable (specific) binding of (+)-ABA to each antibody is indicated by the difference in binding between samples with and without excess unlabelled ABA. MAC252 is a commercially-available, unrelated monoclonal immunoglobulin specific for ABA and is used only as a positive control of specific binding. The data do not allow for a comparison in efficacy between the two. A comparison of MAC252 and the parental 15-I-C5 can be found in Walker-Simmons et al. (1991).
