# Supplementary material for: Kinetic Characterisation of a Single Chain Antibody against the Hormone Abscisic Acid: Comparison with Its Parental Monoclonal
Source: PLoS One. 2016 Mar 29;11(3):e0152148. doi: 10.1371/journal.pone.0152148 (PMC4811560; doi:10.1371/journal.pone.0152148)
Supplement: S4 Fig — (PDF) [file pone.0152148.s005.pdf]

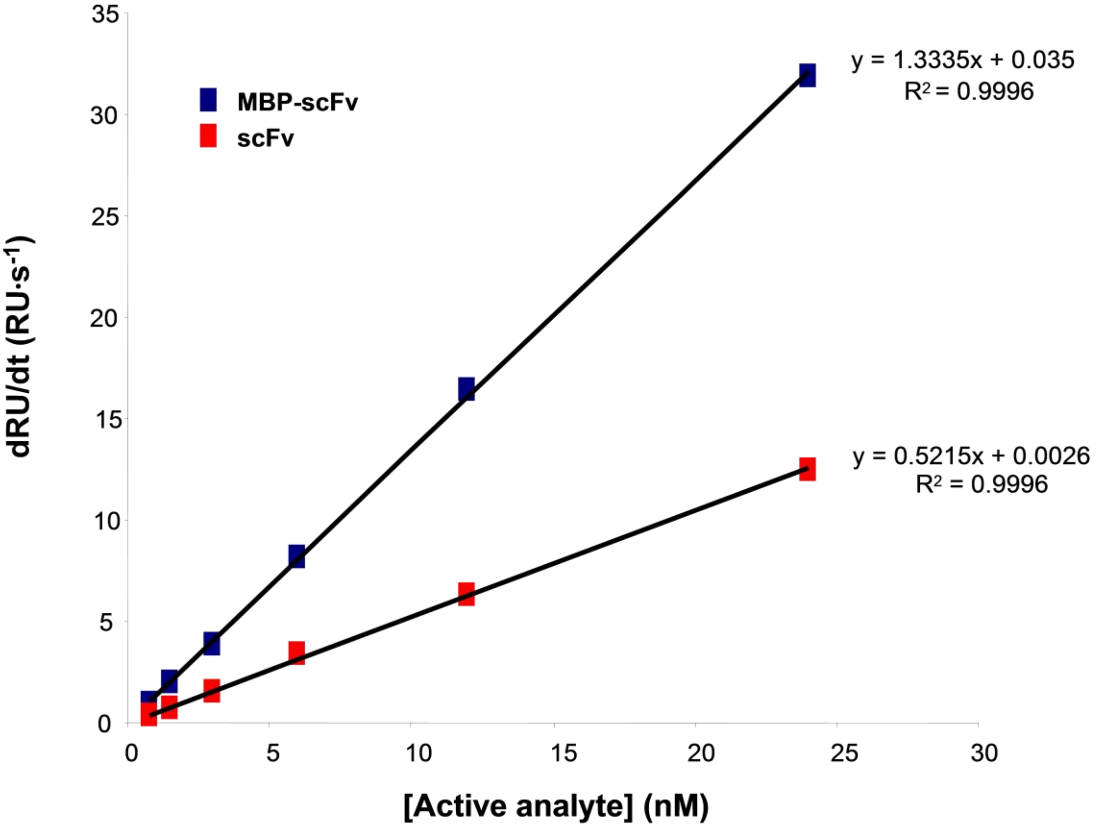

Figure S4. Calibration curve for active protein concentration (Kazemier method). Active protein concentration standards were injected over the surface of a sensor chip saturated with ligand (b-PEG-ABA) and the initial association rate in a 5 s window, 10 s after the start of the injection, was plotted against concentration and fitted to a linear function.
