# Supplementary material for: Kinetic Characterisation of a Single Chain Antibody against the Hormone Abscisic Acid: Comparison with Its Parental Monoclonal
Source: PLoS One. 2016 Mar 29;11(3):e0152148. doi: 10.1371/journal.pone.0152148 (PMC4811560; doi:10.1371/journal.pone.0152148)
Supplement: S5 Fig — (PDF) [file pone.0152148.s006.pdf]

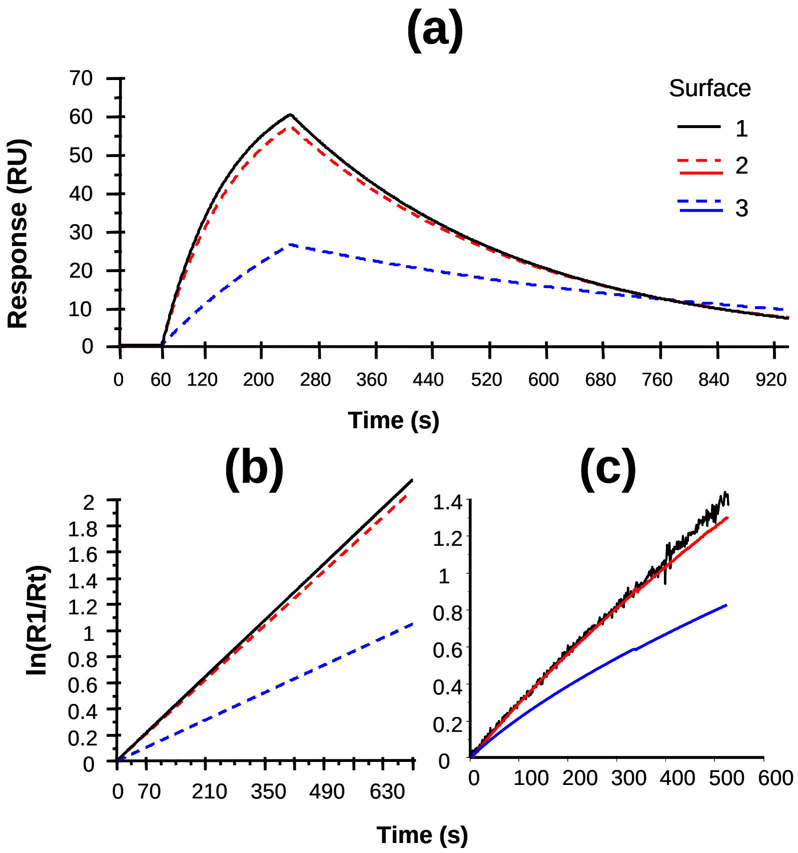

Figure S5. Checking for mass transport limitation; Simulation of the kinetic constants determined on sensor chip surfaces with different ligand densities. **(a)** The simulated sensorgrams (kinetic parameters from data fitted to a simple 1:1 binding model) show that when increasing the ligand density, binding of analyte becomes mass transport limited. **(b)** Dissociation plot of the simulated curves. The slope of the ln(R1/Rt) versus time represents the dissociation rate for that curve. **(c)** Dissociation plot of the dissociation phase of the experimental sensorgrams. The dissociation rate determined on surfaces 2 and 3 is slower than on surface 3 indicating that binding to surfaces 2 and 3 is mass transport limited. Furthermore, a slight curvature can be observed on the plots from surfaces 2 and 3.
