# Supplementary material for: Kinetic Characterisation of a Single Chain Antibody against the Hormone Abscisic Acid: Comparison with Its Parental Monoclonal
Source: PLoS One. 2016 Mar 29;11(3):e0152148. doi: 10.1371/journal.pone.0152148 (PMC4811560; doi:10.1371/journal.pone.0152148)
Supplement: S6 Fig — (PDF) [file pone.0152148.s007.pdf]

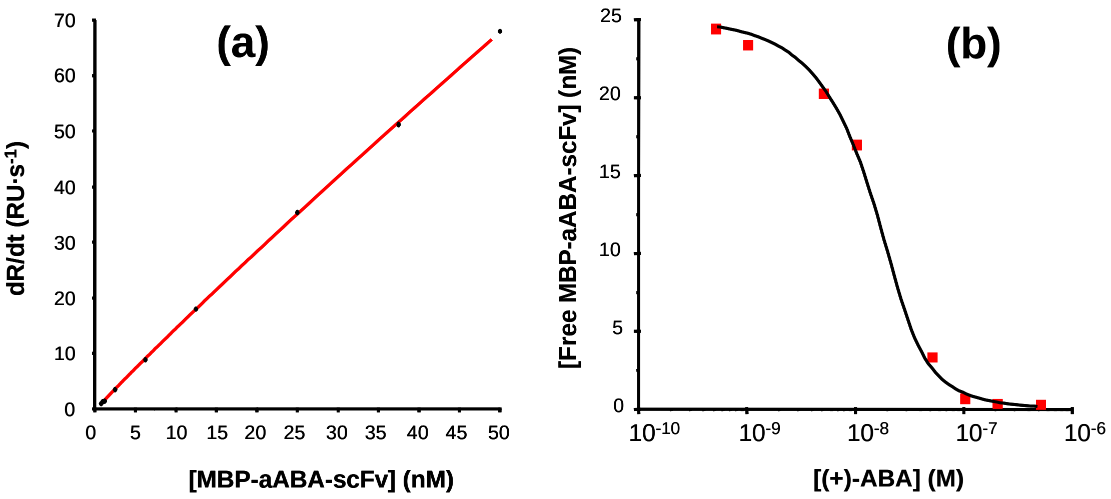

Figure S6. Affinity in solution. (a) A calibration curve was generated by injecting known concentrations of free MBP-antiABA-scFv (0.1 nM – 50 nM) over the surface of a SA sensor chip saturated with b-PEG-ABA, plotting the initial binding rate against concentration and fitting the data points to a 4-parameter equation. **(b)** MBP-antiABA-scFv (25 nM) was mixed with different concentrations of (+)-ABA (0.5 nM, 1 nM, 5 nM, 10 nM, 50 nM, 100 nM, 200 nM, 500 nM), and the mixtures incubated to equilibrium (1 hour at 25°C). The concentration of free MBP-antiABA-scFv in the equilibrium mixtures was then determined from the calibration curve. The concentration of free MBP-antiABA-scFv in the equilibrium mixtures was plotted against (+)-ABA concentration and the data points were fitted to a solution affinity model in BIAevaluate.
