# Supplementary material for: Kinetic Characterisation of a Single Chain Antibody against the Hormone Abscisic Acid: Comparison with Its Parental Monoclonal
Source: PLoS One. 2016 Mar 29;11(3):e0152148. doi: 10.1371/journal.pone.0152148 (PMC4811560; doi:10.1371/journal.pone.0152148)
Supplement: S7 Fig — (PDF) [file pone.0152148.s008.pdf]

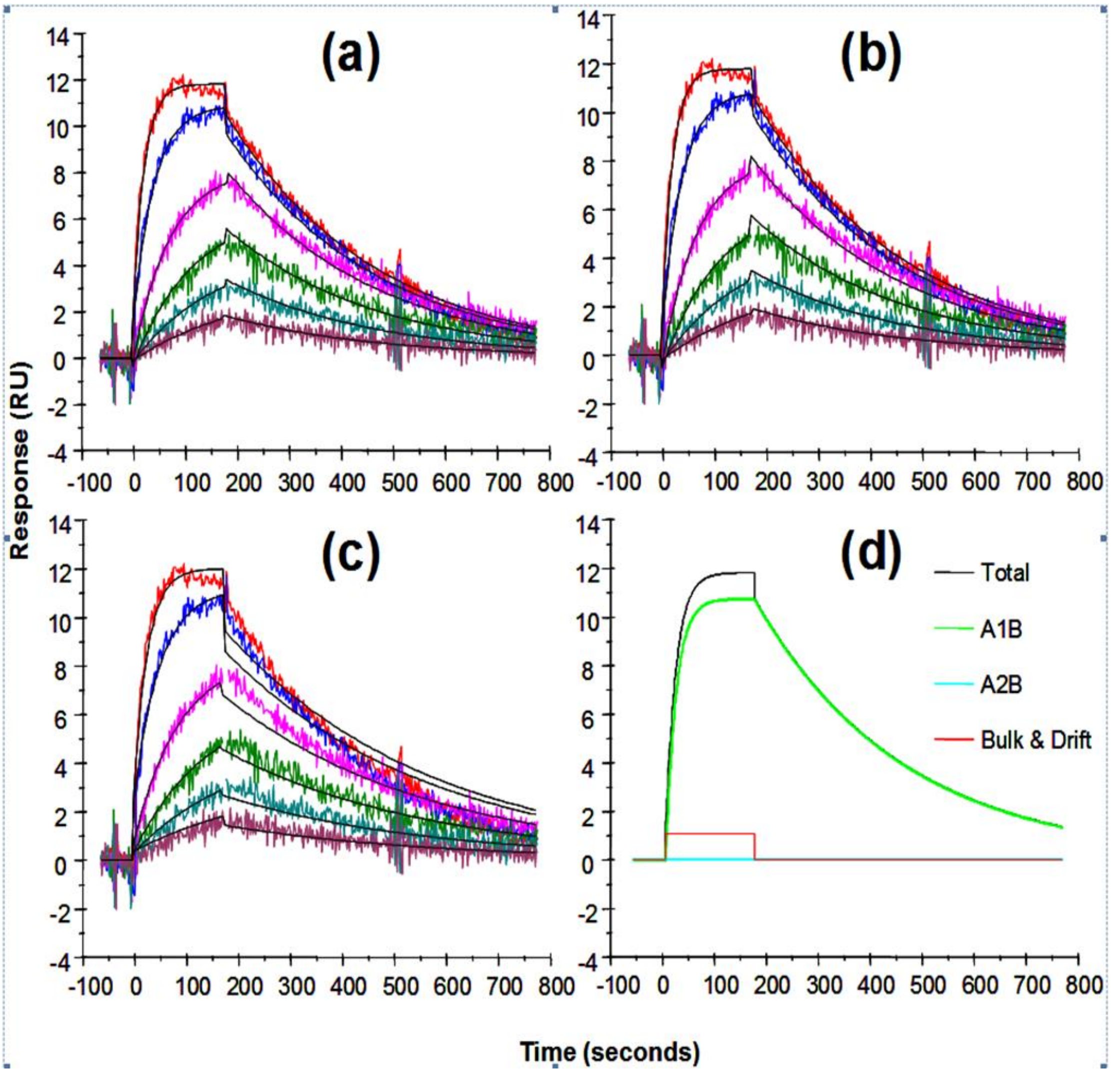

Figure S7. Kinetic analysis of free scFv according to different interaction models. 2-fold dilutions of cleaved aABA-scFv (40-1.25 nM) were used. (a) Sensorgrams were globally fitted to a simultaneous ka/kd 1:1 Langmuir interaction model. (b) SDS-PAGE analysis of the cleaved scFv purified fraction revealed the presence of a secondary band of ≈32.5 kDa. In order to test whether this unidentified band has any binding activity to immobilised ABA, the sensorgrams were globally fitted to a competing reactions (heterogeneous analyte) interaction model. The sizes and proportions of the two bands estimated by SPR (32.6% active scFv) were assumed in the analysis. (c) Sensorgrams were globally fitted to a 2-state interaction model. (d) Simulation of the contribution of the two competing analytes based on the kinetic constants calculated from the competing reactions fit (b).
