# Supplementary material for: Kinetic Characterisation of a Single Chain Antibody against the Hormone Abscisic Acid: Comparison with Its Parental Monoclonal
Source: PLoS One. 2016 Mar 29;11(3):e0152148. doi: 10.1371/journal.pone.0152148 (PMC4811560; doi:10.1371/journal.pone.0152148)
Supplement: S8 Fig — (PDF) [file pone.0152148.s009.pdf]

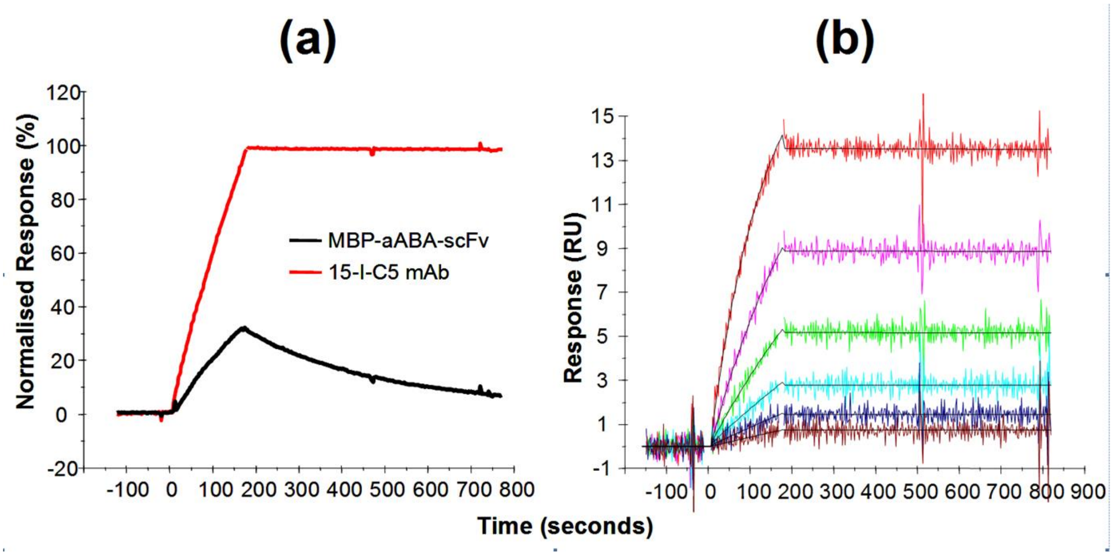

Figure S8. Kinetic analysis of the parental monoclonal immunogobulin 15-I-C5. (a) Overlay of sensorgrams of MBP-aABA-scFv (2nM) and 15-I-C5 parental mAb (2nM). A sensor chip surface with medium ligand density (Rmax ≈ 465RU) was used and the binding response of MBP-aABA-scFv was normalised to the response of 15-I-C5, to which a relative value of 100 was assigned. (b) Concentration series experiment of 15-I-C5 (0.0625-2nM) on a surface with low ligand density (Rmax ≈ 49RU). Sensorgrams were fitted to a bivalent analyte interaction model (fitted curves are shown in black).
