# Supplementary material for: Kinetic Characterisation of a Single Chain Antibody against the Hormone Abscisic Acid: Comparison with Its Parental Monoclonal
Source: PLoS One. 2016 Mar 29;11(3):e0152148. doi: 10.1371/journal.pone.0152148 (PMC4811560; doi:10.1371/journal.pone.0152148)
Supplement: S1 Table — (PDF) [file pone.0152148.s010.pdf]

Table S1.

Yields from expression and purification of anti-ABA scFv in *E. coli.* The best scFv yields were found to be from Origami B cells, grown prior to induction in 100 mM glucose at 37 °C to A_{600} of approximately 0.5, followed by slow induction with 20µM IPTG in the presence of 0.4 M sucrose, for 16 hours at 18 °C,. The yield from the periplasm (pMal-c2x) or cytoplasm (cpMal-c2x) after affinity purification is given per litre of culture.

Fusion protein	Expression vector	Host cell strain	Affinity column	Yield of purified protein (mg·L^{-1})
MBP-scFv	pMal-p2x	Rosetta-gami B	Amylose	0.5
	pMal-c2x	Origami B	Amylose	21.6
	pMal-c2x	BL21	Amylose	3.12
