# Supplementary material for: Kinetic Characterisation of a Single Chain Antibody against the Hormone Abscisic Acid: Comparison with Its Parental Monoclonal
Source: PLoS One. 2016 Mar 29;11(3):e0152148. doi: 10.1371/journal.pone.0152148 (PMC4811560; doi:10.1371/journal.pone.0152148)
Supplement: S2 Table — (PDF) [file pone.0152148.s011.pdf]

Table S2
Determination of the concentration of active MBP-antiABA-scFv.

**Response interval**	**Flow rate** ($\mu l \cdot min^{-1}$)	$\mathbf{L_m}$ **x 10^{-5}** ($m \cdot s^{-1}$)	**dR/dt $_{reg}$** ($RU \cdot s^{-1}$)	χ^2_{reg}	**dR/dt $_{fit}$** ($RU \cdot s^{-1}$)	**MTL**
**I: 100-200 RU**	2	0.32	7.53	0.10	7.48	0.80
	5	0.43	9.53	0.04	9.63	0.75
$[A_{bulk}]$=32.098 nM	10	0.54	11.5	0.04	11.44	0.70
L_r=1.307x10^{-5} $m \cdot s^{-1}$	25	0.73	14.02	0.01	14.03	0.64
χ^2_{fit}=0.0048	50	0.90	16.08	0.00	16.08	0.58
	100	1.1	17.3	0.01	17.28	0.54
**II: 200-300 RU**	2	0.32	7.23	0.03	7.13	0.80
	5	0.43	9.23	0.04	9.27	0.75
$[A_{bulk}]$=30.759 nM	10	0.54	11.1	0.04	11.11	0.71
L_r=1.290x10^{-5} $m \cdot s^{-1}$	25	0.73	13.75	0.04	13.78	0.64
χ^2_{fit}=0.0065	50	0.90	15.85	0.08	15.95	0.58
	100	1.1	17.35	0.09	17.25	0.55
**III: 300-400 RU**	2	0.32	7.08	0.02	7.03	0.78
	5	0.43	9.02	0.04	9.06	0.72
$[A_{bulk}]$=30.791 nM	10	0.54	10.8	0.03	10.77	0.67
L_r=1.1x10^{-5} $m \cdot s^{-1}$	25	0.73	13.21	0.09	13.21	0.60
χ^2_{fit}=0.002	50	0.90	15.08	0.06	15.14	0.54
	100	1.1	16.31	0.08	16.27	0.51
**IV: 400-500 RU**	2	0.32	6.9	0.04	6.87	0.76
	5	0.43	8.74	0.04	8.79	0.70
$[A_{bulk}]$=30.849 nM	10	0.54	10.39	0.01	10.39	0.65
L_r=1.00x10^{-5} $m \cdot s^{-1}$	25	0.73	12.7	0.07	12.65	0.58
χ^2_{fit}=0.002	50	0.90	14.37	0.13	14.41	0.52
	100	1.1	15.41	0.21	15.43	0.49
**V: 500-600 RU**	2	0.32	6.97	0.07	6.77	0.73
	5	0.43	8.43	0.04	8.57	0.65
$[A_{bulk}]$=30.692 nM	10	0.54	9.98	0.07	10.03	0.60
L_r=0.846x10^{-5} $m \cdot s^{-1}$	25	0.73	12	0.04	12.04	0.53
χ^2_{fit}=0.0145	50	0.90	13.56	0.06	13.56	0.46
	100	1.1	14.5	0.07	14.43	0.41
**VI: 600-700 RU**	2	0.32	6.58	0.09	6.51	0.71
	5	0.43	8.11	0.11	8.19	0.66
$[A_{bulk}]$=30.622 nM	10	0.54	9.52	0.07	9.54	0.61
L_r=0.7815x10^{-5} $m \cdot s^{-1}$	25	0.73	11.41	0.04	11.38	0.54
χ^2_{fit}=0.0037	50	0.90	12.81	0.15	12.76	0.48
	100	1.1	13.49	0.11	13.54	0.45

The slopes of sensorgram curves (dR/dt_{reg}) at different flow rates were determined by linear regression in each chosen response interval (I-VI). The χ^2_{reg} values correspond to the fit of the linear regression. The mass transport coefficient (L_m) was calculated according to equation 1. The concentration of MBP-antiABA-scFv ($[A_{bulk}]$) and the coefficient of reaction flux (L_r) were determined by globally fitting the data obtained at different flow rates to equation 2, using dR/dt_{reg} as slopes for each response interval. Ideal slopes (dR/dt_{fit}) were obtained by introducing the calculated L_m, L_r and

A_{bulk} values into equation 2. The measured concentration of *active* MBP-antiABA-scFv, calculated as the average of [A_{bulk}] from each response interval, is 30.97 ± 0.559 nM, which corresponds to 21.8% activity of the total purified protein in the sample (determined by measuring the optical density at 280 nm). The diffusion coefficient for MBP-antiABA-scFv was calculated to be $5x10^{-11}$ $m^2 \cdot s^{-1}$. The concentrations of MBP-antiABA-scFv determined by measuring the UV absorbance at 280 nm was 142nM/10.6$\mu g \cdot ml^{-1}$.
