# Supplementary material for: Kinetic Characterisation of a Single Chain Antibody against the Hormone Abscisic Acid: Comparison with Its Parental Monoclonal
Source: PLoS One. 2016 Mar 29;11(3):e0152148. doi: 10.1371/journal.pone.0152148 (PMC4811560; doi:10.1371/journal.pone.0152148)
Supplement: S3 Table — (PDF) [file pone.0152148.s012.pdf]

Table S3. Determination of the concentration of active proteins by the method of Christensen (1997). Each preparation of antibody was evaluated separately for concentration of active antibody. Thereafter the appropriate concentration calculated from activity measurements was used in all kinetic experiments for that batch of scFv. The data presented are from distinct preparations and are not linked. 'Activity' represents the percentage of the total protein found to be active by SPR. *The commercial monoclonal contained protectants at unspecified concentrations and so we were unable to check the concentration reported by the manufacturers.

**Protein**	**Extinction coefficient ($cm^{-1}\cdot M^{-1}$)**	**A_{280} 0.1%**	**Concentration (nM)**		**Activity (%)**
			**Total (A_{280})**	**Active (SPR)**	
MBP-antiABA-scFv (periplasm)	113470	1.565	141.9	31.0 ± 0.56	21.8±0.39
Cleaved scFv	47120	1.716	142.8	46.6 ± 0.94	32.6±0.66
15-I-C5 mAb	-	-	100*	6.7 ± 0.62	6.7±0.62
