# Supplementary material for: Kinetic Characterisation of a Single Chain Antibody against the Hormone Abscisic Acid: Comparison with Its Parental Monoclonal
Source: PLoS One. 2016 Mar 29;11(3):e0152148. doi: 10.1371/journal.pone.0152148 (PMC4811560; doi:10.1371/journal.pone.0152148)
Supplement: S4 Table — (PDF) [file pone.0152148.s013.pdf]

Table S4. Kinetic and affinity constants of the scFv determined using alternative kinetic models in BIAevaluation. Affinity-purified scFv from the periplasm of Rosetta-gami B *E. coli* cells was injected over sensor chip surfaces with three ligand (b-PEG-ABA) densities. $R_{max} \approx 49$ RU for channel 2, 465 RU for channel 3 and 3100 RU for channel 4.

Chip surface	Fitted model	$R_{max\ fit}$ (RU)	SE (R_{max})	k_a ($M^{-1} \cdot s^{-1}$)	SE (k_a)	k_d (s^{-1})	SE (k_d)	k_t ($RU \cdot M^{-1} \cdot s^{-1}$)	SE (k_t)	K_A (M^{-1})	K_D (M)	χ^2
2	1:1	42.4	0.122	8.67E+05	4.46E+03	2.95E-03	2.28E-05	-	-	2.93E+08	3.40E-09	0.28
2	1:1 with mass transfer	43.7	0.121	8.56E+05	4.47E+03	2.93E-03	2.21E-05	1.59E+08	1.15E+08	2.92E+08	3.42E-09	0.54
2	1:1 with drifting baseline	42.9	0.118	8.65E+05	4.28E+03	3.01E-03	2.19E-05	-	-	2.87E+08	3.47E-09	0.19
3	1:1	455	0.567	7.48E+05	1.55E+03	2.68E-03	7.87E-06	-	-	2.79E+08	3.58E-09	6.17
3	1:1 with mass transfer	447	0.523	8.51E+05	3.17E+03	2.93E-03	1.25E-05	0.73E+09	0.94E+07	2.90E+08	3.44E-09	5.69
4	1:1	3000	5.12	1.95E+05	3.00E+04	1.50E-03	3.52E-05	-	-	1.30E+08	7.68E-09	665
4	1:1 with mass transfer	2950	3.52	2.89E+05	2.25E+04	2.07E-03	1.36E-05	1.13E+09	1.68E+07	1.39E+08	7.17E-09	495
