# Supplementary material for: Kinetic Characterisation of a Single Chain Antibody against the Hormone Abscisic Acid: Comparison with Its Parental Monoclonal
Source: PLoS One. 2016 Mar 29;11(3):e0152148. doi: 10.1371/journal.pone.0152148 (PMC4811560; doi:10.1371/journal.pone.0152148)
Supplement: S5 Table — S5a, Kinetic and equilibrium constants calculated of scFv from cytoplasmic expression in E.coli. S5b. Kinetic and affinity constants calculated for free scFv. (PDF) [file pone.0152148.s014.pdf]

Table S5. Kinetic and equilibrium constants for the scFv expressed and purified from the cytoplasm of E. coli and of free scFv (fusion protein removed).

Table S5a.

Kinetic and equilibrium constants calculated of scFv from cytoplasmic expression in *E.coli*. Data collected at 25°C.

	1:1 Langmuir	2-state
	$A + B \underset{k_{d1}}{\overset{k_{a1}}{\rightleftarrows}} AB$	$A + B \underset{k_{d1}}{\overset{k_{a1}}{\rightleftarrows}} [AB]^* \underset{k_{d2}}{\overset{k_{a2}}{\rightleftarrows}} AB$
k_{a1} ($M^{-1} \cdot s^{-1}$)	9.27E+05	2.73E+06
SE (k_{a1})	3.79E+03	3.90E+04
k_{d1} (s^{-1})	2.63E-03	6.20E-02
SE (k_{d1})	7.03E-06	1.39E-03
k_{a2} (s^{-1})	-	2.36E-02
SE (k_{a2})	-	3.92E-04
k_{d2} (s^{-1})	-	3.75E-03
SE (k_{d2})	-	2.23E-05
K_D (M)	2.84E-09	3.11E-09
χ^2	0.621	0.213

Table S5b. Kinetic and affinity constants calculated for free scFv. The sensorgrams are shown in Supplementary Fig 6.

	Fitted model		
**Parameter**	1:1 Langmuir	Competing reactions	2-state
k_{a1} ($M^{-1} \cdot s^{-1}$)	1.09E+06	1.09E+06	8.87E+05
SE (k_{a1})	8.03E+03	8.03E+03	3.25E+02
k_{d1} (s^{-1})	3.46E-03	3.46E-03	2.54E-03
SE (k_{d1})	1.44E-05	1.44E-05	6.39E-07
k_{a2} ($M^{-1} \cdot s^{-1}$)	-	6.49E+00	1.14E-07*
SE (k_{a2})	-	94.8	1.46E-06
k_{d2} (s^{-1})	-	4.20E-03	5.28E-05
SE (k_{d2})	-	7.81E-03	1.25E-07
K_D (M)	3.17E-09	-	2.85E-09
K_{D1} (M)	-	3.17E-09	-
K_{D2} (M)	-	6.49E-04	-
χ^2	0.141	0.142	0.421

*The unit for this association rate constant is s^{-1}
